# Influence of Aging on Hot Mix Asphalt with the Incorporation of Recycled Concrete Aggregates

**DOI:** 10.3390/ma19020298

**Published:** 2026-01-12

**Authors:** Hugo Alexander Rondón-Quintana, Juan Gabriel Bastidas-Martínez, Saieth Baudilio Chaves-Pabón

**Affiliations:** 1 Facultad de Medio Ambiente y Recursos Naturales, Universidad Distrital Francisco José de Caldas, Bogotá 111711, Colombia; 2 Programa de Ingeniería Civil, Facultad de Estudios a Distancia, Universidad Militar Nueva Granada, Campus de Cajicá, Cajicá 250247, Colombia; juan.bastidas@unimilitar.edu.co (J.G.B.-M.); saieth.chaves@unimilitar.edu.co (S.B.C.-P.)

**Keywords:** recycled concrete aggregates, RCA, hot-mix asphalt, HMA, aging

## Abstract

The aging of asphalt mixture is one of the primary factors influencing the durability and performance of pavements. This study analyzed the influence of short-term (STOA) and long-term (LTOA) aging on hot mix asphalt (HMA) with the incorporation of recycled concrete aggregates (RCAs). The effect of aging on these types of mixtures has not been previously evaluated. HMAs were produced with 0%, 12%, and 21% RCAs (by mass), referred to as HMA Control, HMA RCA12, and HMA RCA21. These replacement percentages correspond to particles ranging between 19 and 12.5 mm (12%) and 19 and 9.5 mm (21%). The Marshall test was employed to determine the optimal asphalt content, followed by indirect tensile strength, resilient modulus, and permanent deformation resistance tests on samples subjected to STOA and LTOA. Overall, the results demonstrate that the incorporation of RCAs could improve the durability of asphalt mixtures by reducing their susceptibility to aging. Specifically, HMA RCA12 exhibited the best balance between stiffness, deformability, and resistance to aging, suggesting a favorable technical potential for its application in sustainable pavements, although additional testing is required to validate its long-term performance. Despite this, high RCA contents may reduce resistance to rutting and moisture damage. The results suggest that the optimal performance is achieved by balancing binder content and aggregate absorption to minimize susceptibility to aging.

## 1. Introduction

### 1.1. Problem Statement and Background

Various studies have used recycled concrete aggregates (RCAs) as substitutes for natural aggregates (NAs) in asphalt mixtures in order to reduce the amounts of construction and demolition waste (CDW) [1]. This practice helps reduce the environmental impacts generated by CDW and provides an environmentally safe disposal method [2]. However, the performance of asphalt mixtures with RCAs is diverse and can be considered contradictory according to the technical literature [3].

Regarding the mechanical behavior of asphalt mixtures with RCAs, the reviewed literature indicates the following: (i) Mixtures with RCAs exhibit higher asphalt binder contents than mixtures with natural aggregates (NAs), primarily due to the higher surface absorption of RCAs [4]. (ii) Most studies report the substitution of NAs with RCAs by mass. However, due to the lower specific gravity of RCAs compared with NAs, a higher quantity of particles is introduced, which increases the asphalt binder content to ensure adequate coating. Therefore, substitution by volume is recommended [5,6]. However, in general, the reported studies meet construction specifications in terms of strength and volumetric composition [2]. (iii) Some studies indicate that the water susceptibility of asphalt mixtures with RCAs improved relative to NAs [7,8,9,10,11], which may be attributed to the increase in asphalt binder content and the contact of water with the adhered cement, activating self-cementing properties and promoting better adhesion between the RCA surface and asphalt binder [7]. In contrast, refs. [12,13,14,15,16] showed that the incorporation of RCAs negatively affected the mixture’s susceptibility, as the high absorption generates greater susceptibility to degradation or failure under wet–dry or freeze–thaw cycles. (iv) Regarding stiffness, some studies report a reduction in the dynamic modulus of mixtures with RCAs compared with the control mixture with NAs [9,17,18,19]. Other studies report a reduction in the resilient modulus (RM) [7,15,20,21,22,23]. Typically, these reductions are proportional to the RCA content incorporated into the mixture, possibly due to the increased optimal asphalt binder content, increased air voids (Avs) in the mixture, and the presence of attached mortar in the RCAs. In contrast, other studies report similar or increased RM values in mixtures with RCA incorporation compared with the control mixture, especially when the RCA source comes from concrete pavements [16,21,24,25]. (v) Regarding permanent deformation resistance, refs. [11,14,18,21,24,26] indicated increases in mixtures with RCAs, reaching up to 40% higher than the control mixture. This behavior is mainly attributed to the greater number of contact points between particles (due to the lower density of RCAs compared with NAs), resulting in better asphalt–aggregate contact and improved particle arrangement in the mixture, due to the angular and cubic shape of RCAs. Despite the good mechanical performance, other studies report contrary results [9,18,19,27], possibly due to high asphalt binder content, Avs, reduced stiffness, and the detachment of the RCA-attached mortar during testing.

In general, the mechanical behavior of asphalt mixtures may depend on the source and origin, chemical and mineralogical composition, surface absorption, physical characteristics, size, and content of RCAs. It also depends on the gradation, type of asphalt mixture, type and content of asphalt binder, among other factors. Some aspects that may lead to contradictory results in the literature could be related to microstructural interactions between the asphalt mastic and aggregate [28], the mineralogical composition of RCAs, and its chemical affinity with asphalt binder [29]. For example, asphalt mixtures with RCAs containing high percentages of silicon dioxide (SiO_2_) may exhibit higher water susceptibility [27].

On the other hand, in the study of asphalt mixtures, it is necessary to evaluate short-term and long-term aging with the aim of reducing in-service pavement distresses such as cracking, stripping, raveling, among others [30]. Short-term aging of asphalt mixtures refers to the oxidation process that occurs during their production at the plant, transportation, and construction (the spreading and compaction of the mixture) [31]. During this process, physicochemical changes occur in the asphalt binder, mainly thermal oxidation due to contact with oxygen, the volatilization of light components, and changes in the asphalt’s microstructure [32]. Over the long term, asphalt binder undergoes aging due to the phenomenon of photo-oxidation caused by ultraviolet (UV) radiation from sunlight, associated with climate effects such as moisture, evaporation, temperature fluctuations, among others, that occur throughout the pavement’s service life [33,34]. Asphalt binder oxidation results in the formation of more molecules and polar functional groups, which reduce mobility, leading to increased viscosity, brittleness, and susceptibility to cracking [35,36]. Although asphalt binder and asphalt mixture aging has been extensively studied, there are few studies addressing this phenomenon in asphalt mixtures with RCAs. Pasandín et al. [37] evaluated in the laboratory the effect of short-term aging time on the properties of a hot mix asphalt (HMA) with RCAs. The mixtures were produced by substituting 0%, 5%, 10%, 20%, and 30% of NAs with RCAs. Prior to compaction, they were kept in an oven for 0, 2, and 4 h at a temperature of 170 °C. Volumetric properties, stiffness, and permanent deformation resistance were studied. The results showed that increasing the aging time of the mixtures with RCAs increased the Av content and stiffness at ambient temperature and reduced the permanent deformation resistance. Another study, conducted by the same authors, aimed to analyze the effect of short-term aging on the stiffness of an asphalt mixture with RCAs [38]. Samples were produced with 0% and 30% RCAs, which were subjected to a 4 h thermal treatment prior to compaction to improve adhesion. The asphalt binder was then extracted using a centrifuge to evaluate its physical and rheological properties, obtaining similar values to those of samples subjected to the Rolling Thin Film Oven Test (RTFOT). Additionally, other results indicated that aging during the thermal treatment increased the stiffness of the mixture, while the presence of RCAs had a lesser influence. Although this study provides interesting results, only the properties of the asphalt binder were evaluated, and not the performance of the asphalt mixture.

### 1.2. Scope and Objective

According to the reviewed literature, numerous studies have analyzed the performance of asphalt mixtures with RCAs with the aim of optimizing the utilization of construction and demolition waste. However, the effect of short-term and long-term aging on these mixtures has not been evaluated, limiting the understanding of their actual in-service behavior. While some studies have focused solely on short-term aging, there is a lack of comprehensive research that simultaneously considers both effects. In this context, the present study proposes an analysis of the combined effect of short-term (STOA) and long-term (LTOA) aging on the mechanical behavior of asphalt mixtures with RCAs. For this purpose, tests of strength under monotonic load (Marshall’s and Indirect Tensile Test—IDT in both dry and wet conditions, including the determination of Tensile Strength Ratio—TSR) and under repeated load (RM and permanent deformation resistance) were conducted. Thus, this work contributes a significant advancement to the knowledge of the performance of asphalt mixtures with RCAs.

## 2. Materials and Methods

**Figure 1:**
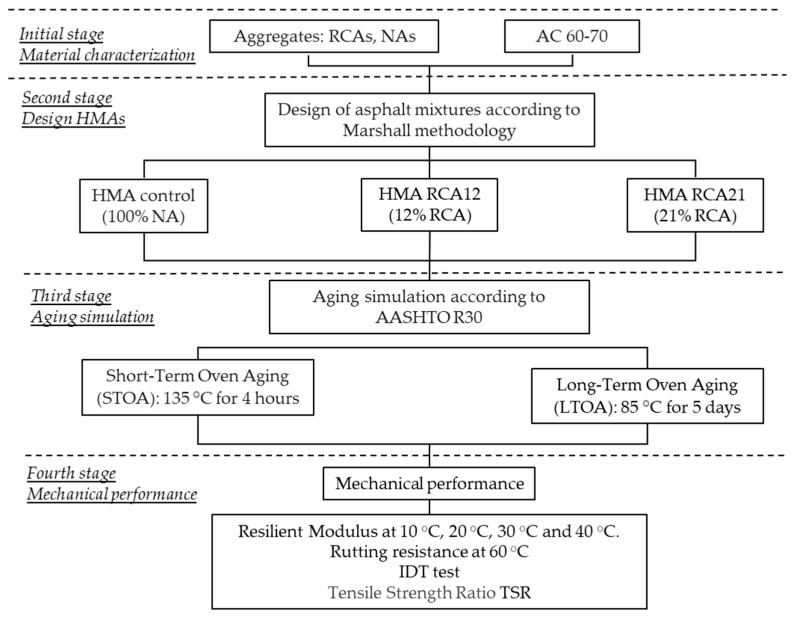
Study methodology.

The experimental phase can be divided into four stages. In the first stage, the materials (RCAs, NAs, asphalt binder) were obtained and characterized. In the second stage, the design of three asphalt mixtures including RCAs and NAs was carried out. Based on previous studies [5,39,40,41], the coarse fraction of NAs was partially replaced by RCAs, as it exhibited a better mechanical performance compared with total replacement. Therefore, in this study, two replacements of the NA fraction by RCAs were made: (i) material passing through a 3/4″ sieve size and retained on the 1/2″ sieve size, i.e., particle size between 19 mm and 12.5 mm, representing 12% of the total aggregate mass; (ii) material passing through a 3/4″ sieve and retained on the 3/8″ sieve size, size between 19 mm and 9.5 mm, representing 21% of the total aggregate mass. These mixtures were named HMA RCA12 and HMA RCA21, respectively. Additionally, a mixture with 100% NAs was prepared and named HMA Control. Particles smaller than RCAs were not incorporated, as the technical literature reports that their use significantly increases the Avs, reduces binder coating on the particles, and may result in mixtures with a high asphalt binder content and poor mechanical performance [27]. In the third phase, using the optimal asphalt content (OAC) determined in the previous design phase, samples of the three mixtures were prepared and subjected to STOA and LTOA processes according to AASHTO R 30. Finally, in the fourth and final phase, Marshall’s tests, resilient modulus, indirect tensile strength, moisture susceptibility, and permanent deformation tests with RCAs were conducted to evaluate the effect of aging. The general methodology of this study is presented in Figure 1.

### 2.1. Materials

The RCAs used in this study were produced from CDW in the city of Bogotá. The NAs correspond to the typical materials used in the production of HMA for pavements. Table 1 presents the physical characterization results of the RCAs and NAs, which meet the quality requirements specified by the INVIAS road construction standard [42].

**Figure 2:**
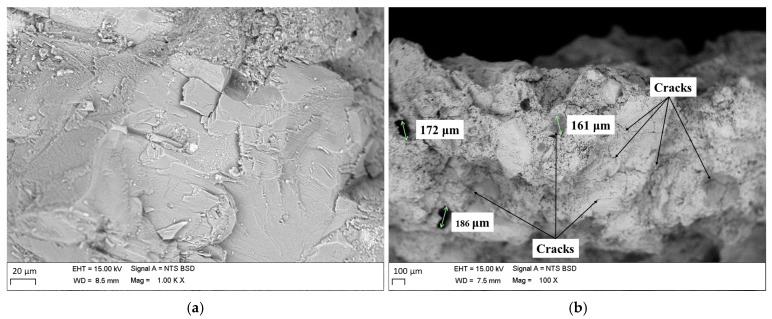
SEM: (**a**) NAs; (**b**) RCAs.

Additionally, RCA and NA samples were analyzed using a JEOL JSM 6700F scanning electron microscope (SEM) (Tokyo, Japan) at 15 kV acceleration voltage under high vacuum, with a working distance of 8.5 mm and a magnification of 5000× (see Figure 2). The chemical composition was also determined by X-ray fluorescence (XRF) and X-ray diffraction (XRD), as presented in Table 2. The RCAs exhibit cracks in the adhered mortar, resulting in a rough surface compared with the NAs. Additionally, circular surface voids with diameters ranging from 160 to 190 µm are observed. The chemical compositions of the RCAs and NAs are similar in terms of the chemical elements. However, the NAs contain higher amounts of iron (Fe) and silica (Si), which may be associated with higher hardness, greater mechanical strength, and better moisture resistance [49,50]. Therefore, HMA with RCAs may exhibit a lower mechanical performance compared with the control mixture (100% NAs). However, RCAs may help improve adhesion due to their higher calcium (Ca) content [51].

The asphalt binder used is an AC 60-70 (penetration grade) or Performance Grade PG 64-22, which is widely used in the production of HMA for pavements in Colombia. Table 3 presents the physical characterization results, which meet the requirements for HMA production according to INVIAS [42].

### 2.2. HMA Design

The asphalt mixture design was carried out using the Marshall test to determine the OAC, following the procedure recommended by ASTM [57] For this purpose, Marshall-type samples comprising 1200 g of mixture were produced in a cylindrical mold approximately 4 inches (10.1 cm) in diameter and 2.5 inches (6.35 cm) in height. The gradation used is presented in Table 4 and corresponds to the average specification for an HMA-19 according to INVIAS [42]

Each sample was compacted with 75 blows per layer using a Marshall hammer (hammer mass of 4.54 kg and drop height of 457.2 mm). Based on the asphalt viscosity curve, the mixing and compaction temperature intervals were defined as 156 ± 4 °C and 148 ± 4 °C, respectively. Before compaction, short-term aging (STOA) was simulated according to AASHTO R30 [58] by subjecting the loose mixture to an oven at 135 °C for four hours. Initial asphalt contents of 4.5%, 5.0%, 5.5%, and 6.0% were used. However, due to the high surface absorption and higher RCA content, an additional asphalt content of 6.5% was evaluated for HMA RCA21. For each sample, mechanical resistance parameters such as stability (S), flow (F), and the S/F ratio were determined. Additionally, volumetric composition parameters were calculated, such as air voids (Avs), Voids in Mineral Aggregate (VMAs), and Voids Filled with Asphalt (VFAs). In total, 39 samples were prepared for the three mixtures, distributed as follows: (i) 12 samples for the HMA Control design; (ii) 12 samples for HMA RCA 12 (4 asphalt contents with 3 repetitions per content); (iii) 15 samples for HMA RCA 21 (5 asphalt contents with 3 repetitions per content).

### 2.3. Short-Term Aging (STOA) and Long-Term Aging (LTOA) Simulation

To simulate the short-term aging process (STOA) according to AASHTO R 30 [58], the loose asphalt mixtures were exposed in an oven at 135 °C for four hours before compaction. After compaction of the HMAs, the samples were subjected to the long-term aging process (LTOA) according to AASHTO R 30 [58]. Each sample was exposed in an oven at 85 °C for 5 days (STOA + LTOA). The LTOA procedure is recommended to simulate the aging that the HMA will undergo between 7 and 10 years of service life of the pavement [31,35,59].

Since both short-term and long-term aging lead to the hardening of the asphalt mixture due to oxidation, volatilization, and other chemical processes, it is expected that parameters such as S, S/F ratio, and RM in LTOA will increase compared with STOA, as widely evidenced in the literature [35,60]. Therefore, to evaluate the effect of aging, the aging ratio or aging increment was established, as shown in Equation (1). This is defined as the ratio between the results of the long-term aged (LTOA) samples and the short-term aged (STOA) samples. Since LTOA generates asphalt mixtures with greater stiffness, the ratio is expected to be greater than 1.0 for the parameters S, S/F, and RM. In other words, the higher this value for these parameters, the greater the susceptibility of the mixture to aging.

(1) $$\mathrm{Aging}\;\mathrm{ratio}=\frac{Value_{LTOA}}{Value_{STOA}}$$

### 2.4. Test

#### 2.4.1. Resilient Modulus (RM)

The RM test was performed using a Universal Testing Machine (UTM-30) (IPC Global, Milan, Italy), applying a repeated load by diametral compression according to UNE-EN [61] to determine the stiffness of the mixtures on Marshall-type samples. The load frequencies used were 2.5 Hz, 5.0 Hz, and 10 Hz at temperatures of 10 °C, 20 °C, 30 °C, and 40 °C. A total of 48 tests were conducted for three types of mixtures, four test temperatures, two aging conditions (STOA/LTOA), and three repetitions per test.

#### 2.4.2. Permanent Deformation Resistance

The determination of the permanent deformation resistance in the asphalt mixtures was performed to evaluate the stiffness under repeated compression load according to UNE-EN [62]. A repeated stress of 100 kPa was applied to Marshall-type samples using a frequency of 10 Hz for 3600 cycles at a temperature of 60 °C. During the test, the average of two axial displacement readings at the ends of the top face of the sample was recorded. Additionally, the displacement velocity of the mixtures (dv) was calculated in μm/min according to Equation (2), where dt_2_ and dt_1_ correspond to the displacements observed at 1800 s (t_1_) and 3600 s (t_2_), respectively. A total of 18 tests were conducted for three types of mixtures, two aging conditions (STOA/LTOA), and three repetitions per test.

(2) $$dv=\frac{d_{t_{2}}-d_{t_{1}}}{t_{2}-t_{1}}$$

#### 2.4.3. IDT Test

The IDT test was conducted according to ASTM [63] with the aim of determining the indirect tensile strength (ITS) by diametral compression of the HMA subjected to STOA and LTOA. Each sample was subjected to a monotonic load with a deformation rate of 48 mm/min until total sample failure. The determination of ITS in kPa was performed according to Equation (3), where P is the maximum load of the test in N; D is the diameter of the sample in mm; and H is the height of the sample in mm.

(3) $$ITS=\frac{2\cdot P}{\pi \cdot D\cdot H}$$

To analyze water susceptibility, the test was performed under both dry and wet conditions using the TSR parameter. In the dry conditions, the samples were kept at 20 °C for 2 h prior to the test. In the wet conditions, the samples were placed in a water bath at 60 °C for 24 h, then subjected to a temperature of 20 °C for 2 h before the test. In this study, the samples were not manufactured with an Av of 7.0 ± 1.0% in order to determine water susceptibility with the same volumetric composition obtained in the mixture design, as this study aims for applicability in tropical countries. The determination of the TSR parameter was performed using Equation (4), where ITSdry and ITSwet correspond to the results of the IDT test in kPa under dry and wet conditions, respectively.

(4) $$TSR=\frac{ITS_{dry}}{ITS_{wet}}\times 100$$

A total of 36 samples were made, corresponding to the following: three types of mixtures, two testing conditions (dry and wet), two aging conditions (STOA/LTOA), and three repetitions per test.

### 2.5. ANOVA

ANOVA statistical analyses, considering a 95% confidence level, were performed with the aim of determining if there were significant variations between the results. For this purpose, three analyses were conducted considering the effect of STOA and LTOA: (i) HMA Control vs. HMA RCA12; (ii) HMA Control vs. HMA RCA21; (iii) HMA RCA12 vs. HMA RCA21.

## 3. Results

### 3.1. Marshall’s Test and Mixtures Design

**Figure 3:**
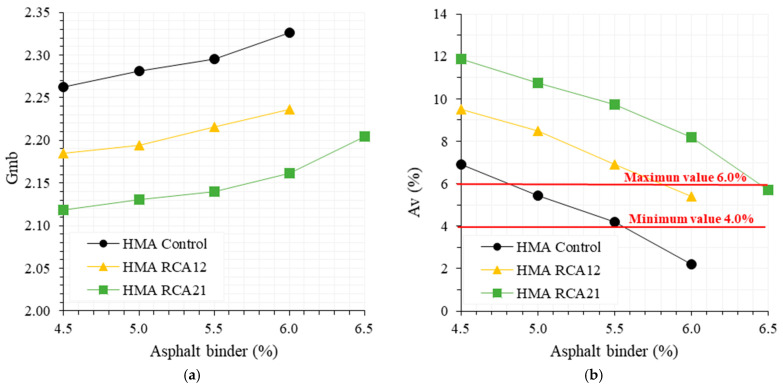

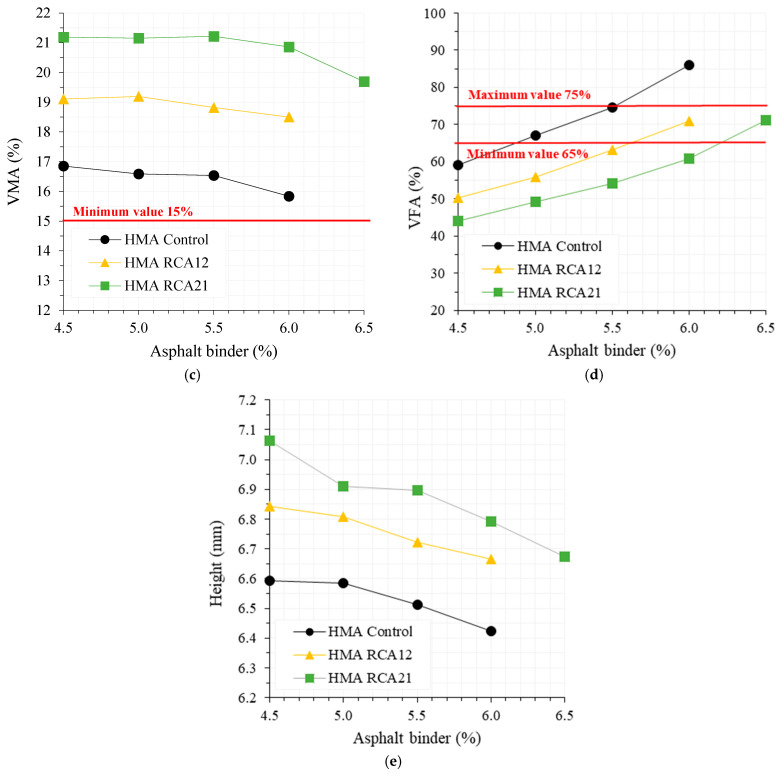
Volumetric composition results: (**a**) Gmb; (**b**) Avs; (**c**) VMAs; (**d**) VFAs; (**e**) height.

The results of the volumetric parameters from the Marshall test (Gmb, Avs, VMAs, VFAs) and the height of the samples for HMA Control, HMA RCA12, and HMA RCA21 are presented in Figure 3. For all asphalt contents analyzed, the results indicate the following: (i) RCAs caused a reduction in the Gmb of the HMA due to the reduced Gs of the RCAs compared with the NAs (see Table 1), attributed to the lower density of the coating mortar. The greatest reduction in Gmm was observed for HMA RCA21, as it contains a higher amount of RCAs. Statistical analysis indicates significant variations in the results (F_T_ > F_0.05_ = 7.70, see Table 5). (ii) RCAs increased Avs and VMAs, and caused a reduction in VFAs in the HMA. This is attributed to the higher absorption of the RCAs, evidenced by the presence of surface voids seen in the SEM analyses. On the other hand, since the substitution was performed by mass and the Gs of RCAs is lower than that of NAs, a higher number of particles was incorporated into the mixture, indicating the need for a higher asphalt binder content to ensure proper coating. Statistical analysis indicates significant variations between the mixtures, as F_T_ > F_0.05_ = 7.70 (see Table 5). (iii) HMA samples with RCAs exhibited higher heights compared with HMA Control, which is attributed to the increased number of RCA particles due to their lower Gs compared with the NAs. Also, the increase in Avs may lead to an increase in the sample volume. Statistical analysis indicates significant variations when comparing the mixtures (see Table 5). (iv) In general, the mixtures meet the quality requirements as outlined in the INVIAS construction standard [42], regarding Avs between 4.0% and 6.0%, minimum VMAs of 15%, and VFAs between 65% and 75%. However, mixtures with RCAs require higher asphalt contents to ensure proper particle coating, primarily due to the surface absorption and the increased number of particles introduced into the mixture during the replacement.

**Figure 4:**
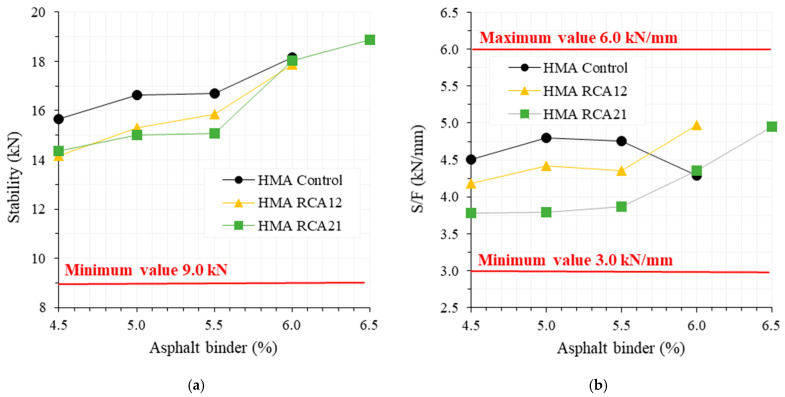
Marshall’s test: (**a**) stability; (**b**) S/F ratio.

The results of mechanical resistance (S and S/F) for the asphalt contents evaluated in the mixtures are presented in Figure 4. It is observed that mixtures with RCAs tend to exhibit lower values of S and S/F for asphalt contents between 4.5% and 5.5%. This behavior can be attributed to the following: (i) the lower mechanical resistance of RCAs compared with NAs (Table 1 shows that RCAs exhibit higher absorption, abrasion resistance in the Los Angeles machine and Micro-Deval, flatness and elongation index, and lower 10% fines compared with NAs, due to the presence of cracks in the mortar adhered to the RCAs; see Figure 2); (ii) the higher porosity of the mixtures with RCAs (Figure 3 shows that mixtures with RCAs exhibit higher Avs and lower VFAs); (iii) insufficient binder content to adequately cover and bond RCAs, which has higher Avs and absorption. However, by increasing the asphalt binder content (from 6%), HMA with RCAs can achieve similar or even higher S and S/F values compared with HMA Control. This is primarily because at this binder content, the coverage and cohesion of RCAs improve, whereas in HMA Control, this content becomes excessive, reducing the material’s stiffness. On the other hand, between 4.5% and 5.5% binder, the mechanical resistance under monotonic load (S/F) tends to be lower with a higher RCA content (HMA RCA21 exhibits the lowest values). This indicates that a higher RCA content may reduce the resistance and stiffness of the mixture. However, by significantly increasing the binder content (6.0% or more), this trend is reversed in HMA RCA21, evidencing the need for higher binder content to achieve an adequate performance. For the evaluated asphalt binder contents, statistical analysis indicates that RCAs do not cause significant variations in the S parameter compared with HMA Control, except for HMA RCA21 with 5.5% asphalt binder (F_T_ > F_0.05_ = 7.70, see Table 6). However, the opposite behavior is observed when analyzing the S/F ratio, which could be related to the deformability of HMA with RCAs. In general, HMA Control, HMA RCA12, and HMA RCA21 meet the quality requirements specified by INVIAS [42] for HMA design, with a minimum S of 9.0 kN and an S/F ratio between 3.0 and 6.0 kN/mm.

Table 7 presents the resistance and volumetric composition parameters that were used to determine the OAC for the mixtures. The OAC was defined based on the criteria established by the construction standard [42]. In general, HMA RCA12 and HMA RCA21 require an increase of 0.5% and 1.0% in the OAC, respectively, compared with HMA Control.

**Figure 5:**
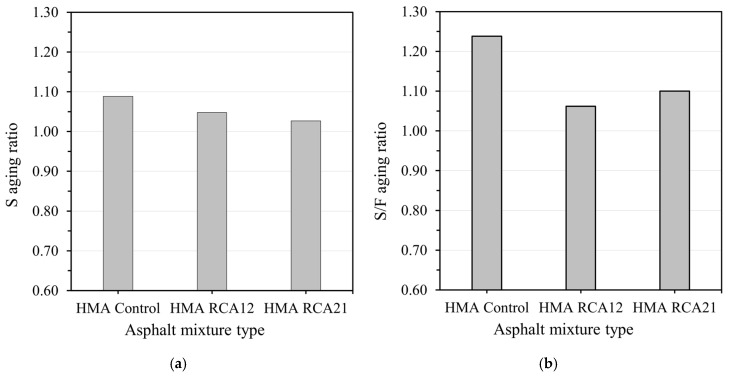
(**a**) Stability and (**b**) S/F aging ratio.

The S and S/F aging ratios of the mixtures are shown in Figure 5. Generally, under moderate oxidative processes, an increase in the S and S/F aging ratios indicates that the mixture is more susceptible to aging. This is because, during oxidation, the viscosity and asphaltene content in the binder increase (hardening and increasing its stiffness, allowing it to withstand more load before failure), while lighter fractions (maltenes) are lost, reducing ductility (the mixture experiences less flow before failure). Based on this, it can be observed that the mixture most susceptible to aging is HMA Control (higher aging ratio). The mixture with the lowest S/F aging ratio was HMA RCA12. This could be due to a better balance between available binder and aggregate absorption in this mixture (a more favorable balance between the structural contributions of RCA and the effective amount of free binder).

### 3.2. Resilient Modulus

**Figure 6:**
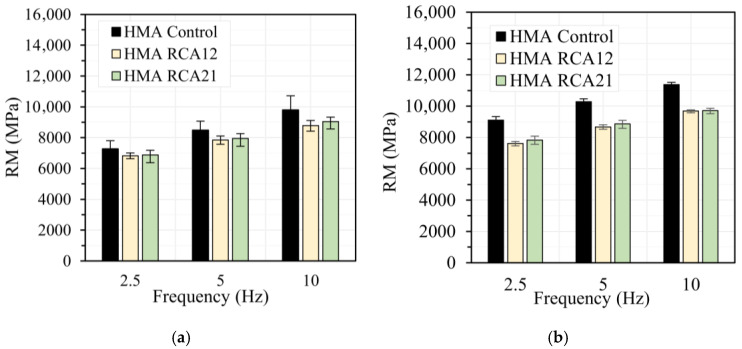
RM test at temperature 10 °C: (**a**) STOA; (**b**) LTOA.

**Figure 7:**
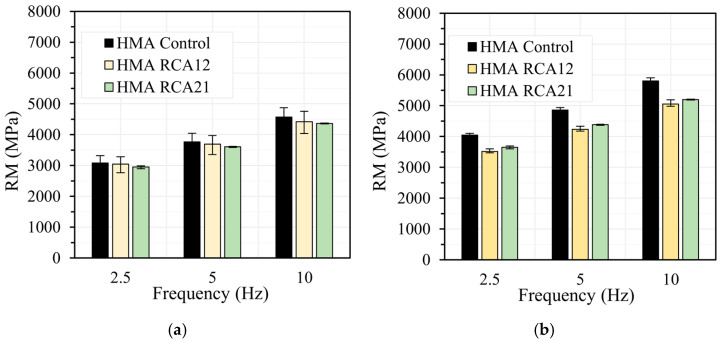
RM test at temperature 20 °C: (**a**) STOA; (**b**) LTOA.

**Figure 8:**
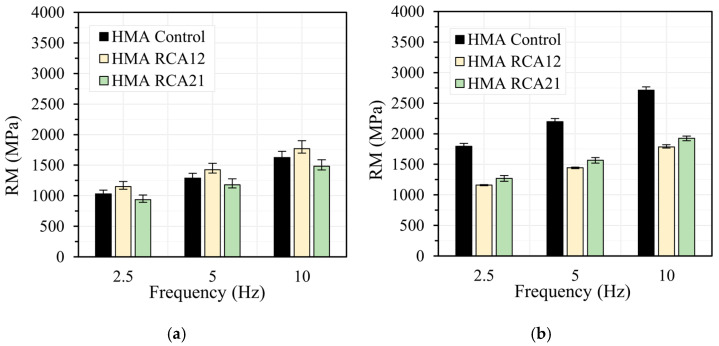
RM test at temperature 30 °C: (**a**) STOA; (**b**) LTOA.

**Figure 9:**
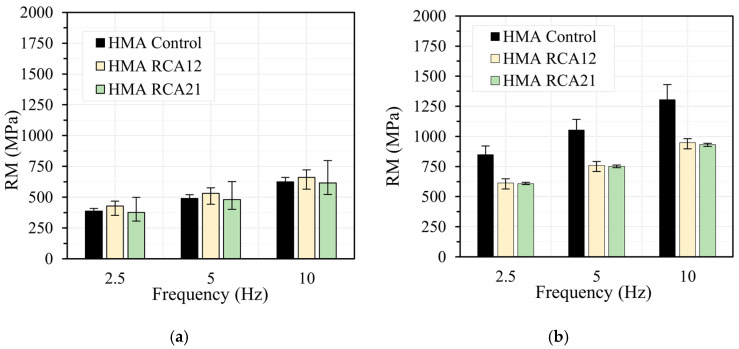
RM test at temperature 40 °C: (**a**) STOA; (**b**) LTOA.

Figure 6, Figure 7, Figure 8 and Figure 9 show the results of the RM test obtained at load frequencies of 2.5 Hz, 5.0 Hz, and 10 Hz, and temperatures of 10 °C, 20 °C, 30 °C, and 40 °C under both STOA and LTOA conditions. In general, the mixtures with RCAs exhibit a slight reduction in RM of approximately 5.0% for HMA RCA12 and 5.5% for HMA RCA21, for all evaluated load frequencies, except for the temperatures of 30 °C and 40 °C with the STOA condition. Statistical analysis indicates that the variations between the results are not significant for the STOA condition, as F_T_ < F_0.05_ = 7.70 (Table 8). These results are consistent with those reported in the literature by Radević et al. [18]. Upon performing LTOA, the mixtures with RCA exhibit lower RM values compared with HMA Control, and these variations are statistically significant (Table 8). These results are attributed to the higher increase in RM for HMA Control due to the aging effect, as thermal oxidation, the volatilization of light asphalt components, polymerization, photo-oxidation, syneresis, and the reorganization of the asphalt microstructure led to the hardening of the asphalt and a greater increase in the mixture’s stiffness [31,62]. These phenomena occurred to a lesser extent in the HMA with RCAs, possibly due to the higher absorption of asphalt components onto the aggregate surfaces.

**Figure 10:**
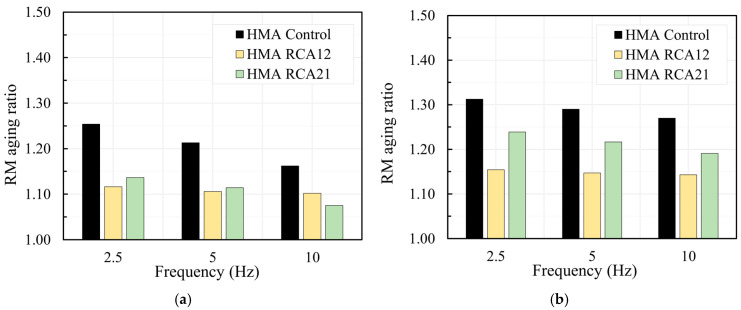

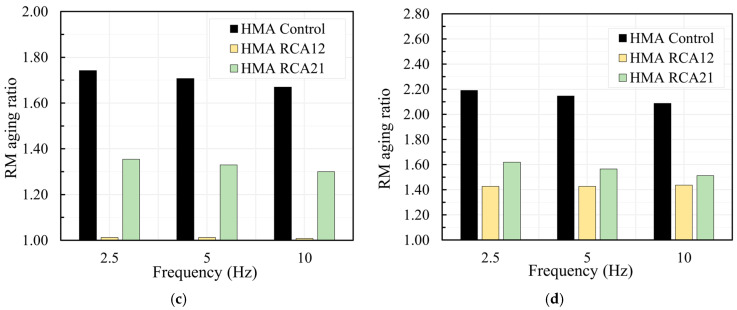
RM Ratio: (**a**) 10 °C; (**b**) 20 °C; (**c**) 30 °C; (**d**) 40 °C.

Figure 10 shows the RM aging ratio results for temperatures of 10 °C, 20 °C, 30 °C, and 40 °C. Mixtures with RCAs exhibited lower susceptibility to aging, as evidenced by the reduction in the RM aging ratio compared with HMA Control, particularly in HMA RCA12. Similarly to the Marshall test, the HMA RCA12 mixture suggests that the effective binder dosage and aggregate absorption reach a better balance. In the HMA RCA21 mixture, the higher Avs and additional absorption likely concentrate some of the binder in the pores of the RCAs, increasing the relative sensitivity to aging. HMA Control, with a lower nominal asphalt binder content (5.5%), shows the highest aging ratio, possibly because the proportional hardening of the binder alters the matrix stiffness more in the absence of the compensatory effects introduced by the RCAs. On the other hand, this asphalt binder content may be insufficient to form a thick enough film to cover the aggregates and resist the aging effects.

### 3.3. Resistance to Permanent Deformation

**Figure 11:**
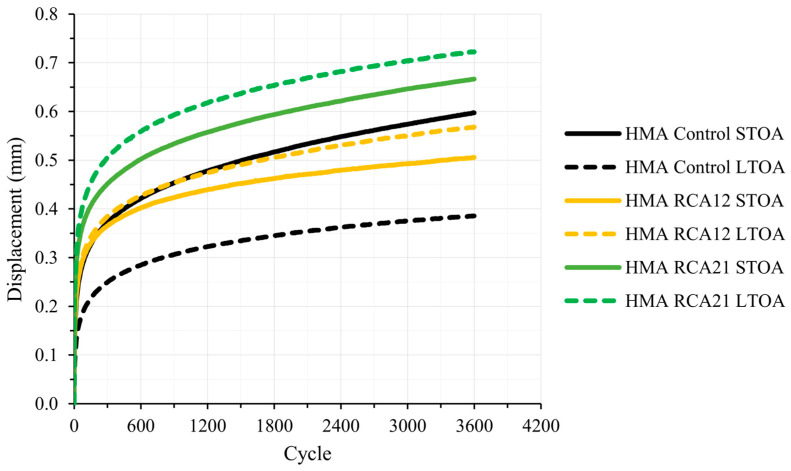
Permanent deformation results at 60 °C.

Figure 11 and Table 9 present the results of the permanent deformation test for HMA Control, HMA RCA12, and HMA RCA21 under STOA and LTOA conditions. Under STOA conditions, the mix that exhibits the highest resistance to permanent deformation is HMA RCA12, followed by HMA Control and HMA RCA21 (defined by the final displacement value). This behavior can be attributed to the enhanced particle accommodation of RCAs in the granular skeleton of the asphalt mix, despite the increase in asphalt binder content and Avs. Similar results have been reported in the literature [3,14,18,26]. However, HMA RCA21 exhibited the opposite behavior. This can be explained by the heterogeneous nature of RCAs: the attached mortar has higher Avs and lower fracture resistance, causing it to micro-crack and detach under repeated loads, which reduces the effective bitumen coating and the intergranular bonding. This was observed in SEM microscopic analysis, where the mortar adhered to the RCAs exhibited cracking, in contrast to the NAs (See Figure 2). Furthermore, in cyclic tests, these factors favor the accumulation of plastic deformations and rutting, even when static or monotonic tests indicate high stiffness. Under LTOA conditions, the mix most resistant to permanent deformation is HMA Control, followed by HMA RCA12 and HMA RCA21 (evidenced by the final displacement and the deformation rate). This behavior is consistent with the results of the RM test. In summary, in terms of long-term permanent deformation accumulation, the mixtures with NAs tend to exhibit lower deformation (greater resistance). However, the lower aging ratio in this mixture indicates that its reduced deformation is driven by an aging-related phenomenon, which is not a reliable indicator of durability. The opposite occurs with the HMA RCA12 mixture, which exhibits the highest aging ratio. These results are consistent with those observed for RM (a higher RM aging ratio tends to translate into a lower displacement aging ratio). Statistical analyses for STOA and LTOA conditions indicate that significant variations exist in the results when considering the final displacement of the test, given that F_T_ > F_0.05_ = 7.70 according to Table 10.

On the other hand, the HMA Control mix exhibits higher resistance to permanent deformation under LTOA conditions compared with STOA. The opposite occurs with the HMA RCA12 and HMA RCA21 mixtures. Long-term aging tends to improve permanent deformation resistance in mixes with NA, but it decreases in mixes with RCAs, due to the higher Avs, absorption, and degradation of the attached mortar, which amplify the negative effects of binder hardening and reduce internal cohesion. This behavior can be attributed to the following: (i) the presence of surface voids in the RCAs may have led to the encapsulation of the lighter asphalt fractions during short- and long-term aging, contributing to reducing asphalt aging; (ii) the higher asphalt binder content and Avs in the RCA mixes may have generated greater deformability during testing; (iii) during the LTOA process, the slight degradation or micro-cracking of the mortar attached to the RCAs may have occurred, possibly causing particle reorganization during loading cycles in the test. In practical terms, HMA Control could improve its resistance to rutting over the long term, but this increased stiffness may increase brittleness and the risk of cracking due to fatigue or thermal stresses. In the case of HMA RCA12, an initially good resistance to rutting is observed; however, the performance decline after LTOA indicates a susceptibility to aging-related changes (depending on the amount of effective binder remaining). HMA RCA21 experiences the worst performance under all relative conditions.

There is a direct relationship between the final displacement and the deformation rate. In other words, the mixture that exhibits greater resistance to permanent deformation is characterized by the lowest values. In this regard, HMA RCA12 and the control HMA mixture show higher resistance in STOA and LTOA, respectively. However, in terms of aging resistance, the control mixture shows a weaker performance (lower displacement and dv aging ratios).

**Figure 12:**
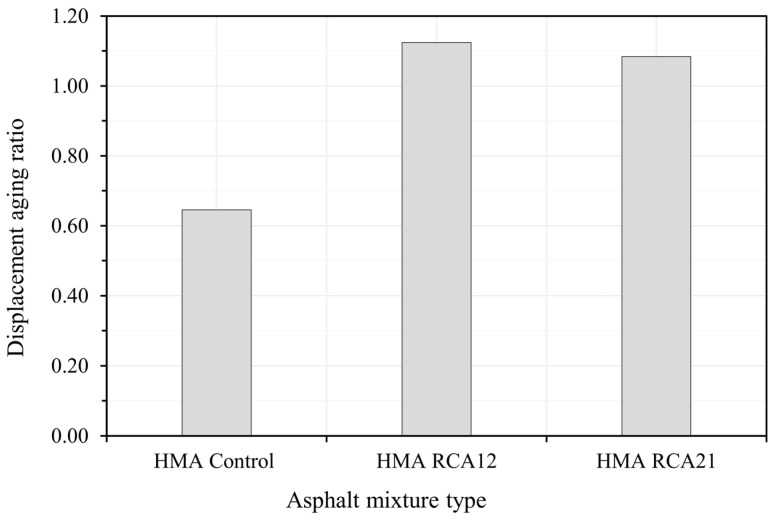
Displacement aging ratio.

Figure 12 presents the displacement aging ratio for HMA Control, HMA RCA12, and HMA RCA21. After LTOA, the mix without RCAs (HMA Control) showed a significant reduction in maximum strain, indicating that prolonged aging increased its resistance to rutting, likely due to binder hardening and volumetric adjustment. In contrast, the mixes with RCAs (HMA RCA12 and HMA RCA21) exhibited increases in maximum strain after LTOA, suggesting that aging promoted mechanisms favoring creep (e.g., lower effective binder content due to absorption in the RCAs, higher Avs, and the possible degradation of the adhered mortar), leading to a reduction in rutting resistance.

### 3.4. IDT Test and TSR

**Figure 13:**
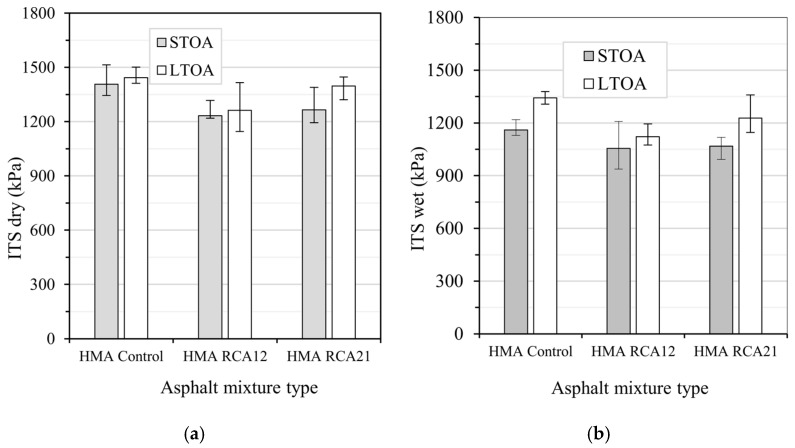
IDT test: (**a**) dry; (**b**) wet.

The results of the IDT test under dry and wet conditions, considering STOA and LTOA, are presented in Figure 13. The mixes with RCA showed lower ITS compared with HMA Control for both STOA and LTOA conditions. Under dry conditions, these reductions are approximately 19.5% and 16.4% for HMA RCA12 and HMA RCA21, respectively, for STOA. For the LTOA condition, these percentages are 12.5% and 3.4%, respectively. Under wet conditions, for STOA, the reductions are approximately 9.0% and 7.9% for HMA RCA12 and HMA RCA21, respectively. In LTOA, these values correspond to 16.5% and 8.6%, respectively. However, statistical analyses indicate that the variations are not significant, as F_T_ > F_0.05_ = 7.70, except when analyzing HMA RCA21 (See Table 11). Despite having higher binder contents, the reduction in ITS in the mixes with RCAs can be attributed to the following: (i) their higher Av content (Figure 3 and Table 7); (ii) the lower resistance of RCA particles (Table 1); (iii) the formation of thinner asphalt binder films around the aggregates, affecting internal adhesion (part of the binder is absorbed into the RCA pores, reducing adhesion); (iv) the presence of attached mortar with calcium hydroxides, amorphous silica, etc., may result in poor adhesion between the binder and the aggregate, promoting interfacial failures. These results are consistent with the literature, where similar behavior in RCA mixes shows a lower tensile strength compared with HMA Control [7,13,16,23].

**Figure 14:**
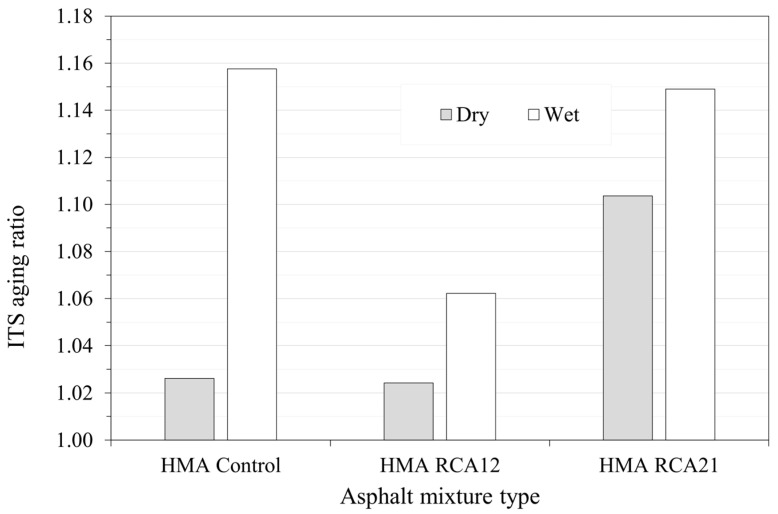
ITS aging ratio.

Figure 14 presents the results of the ITS aging ratio to evaluate the effect of aging on asphalt mixtures. In theory, ITS aging ratio values should be lower than 1.0, as binder hardening through oxidation reduces its deformation and adhesion capacity, causing fractures to occur more easily since the mixes become more brittle. However, in this study, the ITS aging ratio values are greater than 1.0. This behavior can be attributed to an increase in the stiffness and cohesion of the asphalt matrix due to the increase in binder viscosity and possible volumetric adjustment during the LTOA process, which reduces voids and improves internal compaction. In the mixes with RCAs, partial binder absorption and additional hardening of the attached mortar may have contributed to consolidating the internal structure, limiting the negative effect of aging on resistance. Therefore, the observed increase in ITS does not necessarily indicate lower durability, but rather a stiffer and more cohesive state of the mix within the aging range applied (aging tends to reinforce the internal structure and cohesion, meaning the asphalt matrix may have rigidified without degrading its mechanical integrity). This increase in ITS after aging should be interpreted as an improvement in immediate structural stability, but not necessarily as an increase in the overall durability of the material. Additionally, these mixtures may possibly exhibit increased brittleness.

On the other hand, Figure 14 shows a trend similar to that reported in the Marshall and RM tests. The HMA RCA12 mix exhibits the least sensitivity to aging in terms of indirect tensile strength, suggesting an optimal balance between effective binder and aggregate absorption. These improvements in ITS after LTOA should be interpreted with caution: they indicate less strength loss due to aging, but do not rule out an increase in brittleness (greater susceptibility to cracking, according to the high cracking of the adhered mortar reported in the SEM analysis; see Figure 2). Therefore, it is recommended that future work determine additional test parameters such as peak deformation, fracture energy analysis, among others.

**Figure 15:**
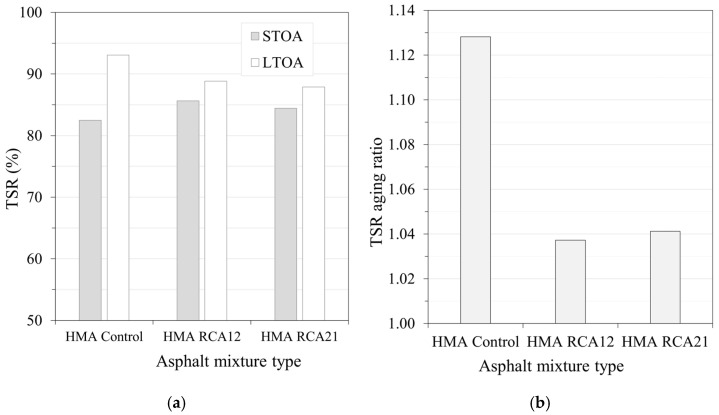
Results of (**a**) TSR and (**b**) TSR aging ratio.

Figure 15a presents the TSR results considering the effects of STOA and LTOA for HMA Control, HMA RCA12, and HMA RCA21. The TSR parameter allows for the evaluation of the effect of water susceptibility, indicating that higher TSR values are associated with greater mix adhesion and resistance to moisture damage. Under STOA conditions, HMA RCA12 and HMA RCA21 showed an increase of approximately 3.2% and 1.9%, respectively, compared with HMA Control. This behavior can be attributed to the increased asphalt binder content and the greater and better penetration of the binder into the surface voids of the RCAs during the short-term aging period before compaction. These results are consistent with those reported in the literature [8,11,26]. On the other hand, under LTOA conditions, the response is the opposite. HMA RCA12 and HMA RCA21 showed a decrease in TSR values by approximately 4.2% and 5.2%, respectively, compared with HMA Control. In Figure 15b, the TSR aging ratio was >1.0 for all three mixes, indicating that long-term aging did not decrease, but rather improved or maintained resistance retention against moisture damage. HMA Control showed the greatest relative increase, while the RCA mixes showed smaller changes, indicating greater stability. These variations can be attributed to the combined effects of moderate oxidation (increased polarity/adhesion), the removal of residual moisture, and volumetric readjustment during LTOA. In summary, the mix with the lowest overall susceptibility is HMA RCA12, while the most susceptible tends to be HMA Control.

## 4. Summary and Discussion

The main objective of this study is to evaluate the effect of short-term and long-term aging on the mechanical behavior performance of an HMA with RCAs. To this end, the mix design was carried out using the Marshall method, incorporating 0%, 12%, and 21% RCAs (by mass), which were labeled as HMA Control, HMA RCA12, and HMA RCA21. Subsequently, mixtures were produced and subjected to the STOA and LTOA procedures to simulate aging. IDT tests were conducted under both dry and wet conditions to assess water susceptibility. Additionally, repeated load tests, such as RM and permanent deformation resistance, were performed. To evaluate the mechanical performance, the HMA was rated on a scale from 1 to 3 (see Table 12 and Table 13), where 1 corresponds to the highest value and 3 to the lowest value. In the parameters, 1 indicates higher resistance, except for permanent deformation where the reverse is true (3 is the most susceptible). In the aging ratio, 1 indicates higher susceptibility to aging. The results indicate the following: (i) The mixes with RCAs exhibited higher asphalt binder contents compared with the mix with NAs. These increases correspond to 0.5% for HMA RCA12 and 1.0% for HMA RCA21, relative to HMA Control. These results are primarily attributed to RCA absorption and the reduction in specific gravity, which creates a larger surface area to be covered by the greater number of RCA particles incorporated into the mix. Additionally, the increase in OAC in the HMA with RCAs led to an increase in S and S/F relative to the control mix. This behavior can be evidenced by the trends of 1.0 and 2.0 values in the Marshall test ratings. (ii) In general, the mixes with RCAs under both STOA and LTOA conditions exhibited slight reductions in ITS (dry and wet conditions), increased RM, and an increase in the TSR parameter compared with HMA Control. This is evidenced by the trends of 1.0 values for ITS and RM, and 3.0 for TSR in HMA Control. However, statistical analysis trends indicated that these variations are not significant. Even Table 14 shows that most of the coefficients of variation (COVs) are small (<10%) across the results obtained from the different tests, indicating low dispersion and good homogeneity (high consistency, good repeatability, and low experimental uncertainty). These results can be attributed to the increased asphalt binder content relative to HMA Control. (iii) Permanent deformation resistance results under STOA conditions indicated that HMA RCA12 performed better than HMA Control. However, HMA RCA21 showed the opposite behavior. Therefore, the amount of RCAs influences the mechanical behavior of the mix. (iv) The mixtures with RCAs showed lower susceptibility to aging, defined by the reduction in ITS aging ratio, TSR aging ratio, and displacement aging ratio compared with HMA Control. That is, HMA Control exhibited higher susceptibility to aging, as the general trend of the ratings indicates 1.0 values for all evaluated parameters, except for the ITS dry value. Thus, it can be demonstrated that the presence of RCAs could result in the lower variability of mechanical parameters over the pavement’s service life due to the effects of long-term aging.

An optimum or slightly higher asphalt binder content, together with the chemical and mineralogical characteristics of the RCAs, could contribute to improving the aging resistance of asphalt mixtures. A higher effective binder content helps compensate for absorption within the RCAs, maintaining adequate film thickness and reducing binder hardening during service. Additionally, the adhered cement paste contains alkaline compounds such as calcium hydroxide and calcium carbonate, which may partially neutralize acidic oxidation products and provide adsorption sites for polar components, thus stabilizing the asphalt–aggregate interface. The porous and heterogeneous microstructure of the RCAs allows for the partial absorption of the binder, retaining light fractions that limit volatilization, while its rough surface enhances mechanical interlocking and adhesion, mitigating interfacial microcracking. Furthermore, the carbonate minerals contribute to a more uniform thermal distribution and reduced oxygen diffusion. Altogether, these mechanisms may retard oxidative and physical aging, improving the long-term durability of mixtures incorporating RCAs.

## 5. Conclusions

Based on the results obtained, the following can be concluded:

▪ The mixtures with RCAs show higher resistance under a monotonic load in the Marshall test (S, S/F ratio) compared with HMA Control. However, from a statistical standpoint, these increases in resistance are not significant. Furthermore, these mixes show lower susceptibility to aging (especially HMA RCA12).
▪ As the mix most susceptible to aging in the RM tests, HMA Control exhibits the highest RM values under LTOA conditions. Under STOA conditions, the changes in RM for the three mixes are not statistically significant.
▪ The least resistant mix to rutting is HMA RCA21. Therefore, its use is not recommended in high-temperature climates.
▪ Despite being the mix most susceptible to aging in the IDT tests, HMA Control exhibits the highest ITS values and tends to be the most resistant to moisture damage. However, its higher stiffness and lower deformation capacity could translate into an inferior performance under low-temperature and fatigue conditions.
▪ In all tests, the mix least susceptible to aging is HMA RCA12, and the most susceptible tends to be HMA Control. Thus, the incorporation of RCAs contributes from a technical perspective, as the reduction in aging susceptibility provides greater durability and a better performance for a pavement. However, additional tests should be conducted to validate the performance of HMA with RCAs.
▪ There is a need to increase the binder content in HMA Control to improve its resistance to aging.
▪ The results indicate the existence of an optimal balance between binder content and aggregate absorption, where the asphalt mixes exhibit the lowest susceptibility to aging.
▪ As widely reported in the literature, HMA mixes with RCAs require the incorporation of a higher asphalt binder content to ensure compliance with the quality requirements specified by Colombian standards.
▪ In general, mixtures with RCAs exhibited a slight reduction in stiffness, as measured by the RM and in indirect tensile strength under dry and wet conditions compared with the control HMA. Marshall stability and permanent deformation resistance increased with the inclusion of RCAs. However, when subjected to long-term aging, mixtures with RCAs showed lower susceptibility, potentially improving durability.

These results are valid for dense mixtures composed of the materials described in the characterization (RCAs, NAs, and AC 60-70), as well as for RCA contents of 12% and 21% (percentage with respect to the total aggregate mass). The following are recommended for future work: (i) conduct fatigue resistance tests; (ii) assess performance at low temperatures; (iii) validate in the field with Full-Scale Pavement Tests or Accelerated Pavement Testing (APT); (iv) perform economic and environmental impact analysis; (v) conduct studies considering the influence of increasing the COA in the Marshall mix design methodology to mitigate aging-related effects.

## Figures and Tables

**Table 1:** Physical characterization tests of RCAs and NAs.

Test	Method	RCAs	NAs	Requirement
Abrasion in Los Angeles machine	[43]	24.94	18.7	<25%
Abrasion in Micro-Deval	[44]	17.6	8.2	<20%
10% of fines (dry resistance)	[45]	143.5	227.5	>100 kN
Flattening Index (%)	[46]	29.1	18.7	---
Elongation Index (%)	[46]	22.9	14.6	---
Fractured particles, 1 side (%)	[47]	92.1	95.2	>85%
Specific Gravity of coarse aggregate	[48]	2.46	2.61	---
Bulk Specific Gravity	[48]	2.15	2.54	---
Coarse aggregate absorption (%)	[48]	6.82	1.85	---

**Table 2:** Composition of NAs and RCAs in SEM.

Material	C	O	Al	Si	Ca	Fe	Others	Total
NA	16.27	37.31	2.51	18.06	12.39	9.20	4.26	100
RCA	10.42	53.90	2.48	7.15	23.40	3.74	---	100

**Table 3:** Physical characterization tests of AC 60-70.

Test	Method	Result	Requirement
Neat asphalt			
Penetration (0.1 mm)	[52]	65	60–70
Softening point (°C)	[53]	49.6	48–54
Penetration Index	[54]	−0.67	−1.2–0.6
Viscosity at 60 °C (P)	[55]	2539	>1500
Ductility (cm)	[56]	100+	>100
Rolling Thin Film Oven Test—RTFOT			
Loss of mass (%)		0.567	<0.8
Penetration of the residue in reference to the original (%)	[52]	52.9	>50
Increase softening point (°C)	[53]	6.8	<9.0

**Table 4:** Particle size distribution for HMAs.

**Size (mm)**	19.0	12.5	9.5	4.75	2.0	0.425	0.18	0.075	Bottom
**Passing (%)**	100.0	87.5	79.0	57.0	37.0	19.5	12.5	6.0	0.0
**Retained (%)**	0.0	12.5	8.5	22.0	20.0	17.5	7.0	6.5	6.0

**Table 5:** ANOVA for the significance of Gmb, Avs, VMAs, VFAs, and height for HMA.

Analysis	4.5% Asphalt Binder			5.0% Asphalt Binder			5.5% Asphalt Binder			6.0% Asphalt Binder		
	F_T_	*p*-Value	Sig	F_T_	*p*-Value	Sig	F_T_	*p*-Value	Sig	F_T_	*p*-Value	Sig
Gmb												
HMA Control vs. HMA RCA12	412.17	3.48 × 10^−5^	Yes	359.80	4.55 × 10^−5^	Yes	373.53	4.22 × 10^−5^	Yes	1447.35	2.85 × 10^−6^	Yes
HMA Control vs. HMA RCA21	2570.57	9.06 × 10^−7^	Yes	1772.56	1.90 × 10^−6^	Yes	865.45	7.95 × 10^−6^	Yes	934.56	6.82 × 10^−6^	Yes
HMA RCA12 vs. HMA RCA21	230.71	1.10 × 10^−4^	Yes	142.74	2.81 × 10^−4^	Yes	223.92	1.16 × 10^−4^	Yes	198.75	1.47 × 10^−4^	Yes
Avs												
HMA Control vs. HMA RCA12	271.10	7.97 × 10^−5^	Yes	249.98	9.35 × 10^−5^	Yes	250.67	9.30 × 10^−5^	Yes	1024.95	5.67 × 10^−6^	Yes
HMA Control vs. HMA RCA21	1777.67	1.89 × 10^−6^	Yes	1251.48	3.81 × 10^−6^	Yes	620.00	1.54 × 10^−5^	Yes	681.55	1.28 × 10^−5^	Yes
HMA RCA12 vs. HMA RCA21	170.17	1.99 × 10^−4^	Yes	103.89	5.22 × 10^−4^	Yes	171.93	1.95 × 10^−4^	Yes	152.11	2.48 × 10^−4^	Yes
VMAs												
HMA Control vs. HMA RCA12	256.29	8.90 × 10^−5^	Yes	236.90	1.04 × 10^−4^	Yes	234.65	1.06 × 10^−4^	Yes	963.57	6.42 × 10^−6^	Yes
HMA Control vs. HMA RCA21	1693.43	2.08 × 10^−6^	Yes	1189.32	4.22 × 10^−6^	Yes	587.48	1.72 × 10^−5^	Yes	644.72	1.43 × 10^−5^	Yes
HMA RCA12 vs. HMA RCA21	163.61	2.15 × 10^−4^	Yes	99.21	5.71 × 10^−4^	Yes	164.91	2.12 × 10^−4^	Yes	145.22	2.72 × 10^−4^	Yes
VFAs												
HMA Control vs. HMA RCA12	341.10	5.06 × 10^−5^	Yes	312.57	6.01 × 10^−5^	Yes	242.22	9.95 × 10^−5^	Yes	953.90	6.55 × 10^−6^	Yes
HMA Control vs. HMA RCA21	2239.46	1.19 × 10^−6^	Yes	1543.50	2.51 × 10^−6^	Yes	669.69	1.32 × 10^−5^	Yes	1009.67	5.85 × 10^−6^	Yes
HMA RCA12 vs. HMA RCA21	162.36	2.19 × 10^−4^	Yes	99.97	5.62 × 10^−4^	Yes	189.29	1.62 × 10^−4^	Yes	190.24	1.60 × 10^−4^	Yes
Height												
HMA Control vs. HMA RCA12	41.24	3.02 × 10^−3^	Yes	124.71	3.66 × 10^−4^	Yes	21.06	1.01 × 10^−2^	Yes	168.05	2.04 × 10^−4^	Yes
HMA Control vs. HMA RCA21	135.41	3.12 × 10^−4^	Yes	65.36	1.27 × 10^−3^	Yes	61.71	1.42 × 10^−3^	Yes	44.26	2.65 × 10^−3^	Yes
HMA RCA12 vs. HMA RCA21	22.10	9.30 × 10^−3^	Yes	6.48	6.36 × 10^−2^	No	44.25	2.65 × 10^−3^	Yes	5.83	7.33 × 10^−2^	No

**Table 6:** ANOVA for the significance of S and S/F for HMA.

Analysis	4.5% Asphalt Binder			5.0% Asphalt Binder			5.5% Asphalt Binder			6.0% Asphalt Binder		
	F_T_	*p*-Value	Sig	F_T_	*p*-Value	Sig	F_T_	*p*-Value	Sig	F_T_	*p*-Value	Sig
**S**												
HMA Control vs. HMA RCA12	2.99	1.59 × 10^−1^	No	4.14	1.12 × 10^−1^	No	4.19	1.10 × 10^−1^	No	0.20	6.79 × 10^−1^	No
HMA Control vs. HMA RCA21	2.65	1.79 × 10^−1^	No	6.80	5.96 × 10^−2^	No	18.79	1.23 × 10^−2^	Yes	0.04	8.55 × 10^−1^	No
HMA RCA12 vs. HMA RCA21	0.13	7.36 × 10^−1^	No	0.30	6.14 × 10^−1^	No	4.58	9.90 × 10^−2^	No	0.04	8.54 × 10^−1^	No
**S/F**												
HMA Control vs. HMA RCA12	2.39	1.97 × 10^−1^	No	2.07	2.24 × 10^−1^	No	9.03	3.97 × 10^−2^	Yes	10.70	3.08 × 10^−2^	Yes
HMA Control vs. HMA RCA21	8.03	4.72 × 10^−2^	Yes	12.25	2.49 × 10^−2^	Yes	41.95	2.93 × 10^−3^	Yes	0.07	8.05 × 10^−1^	No
HMA RCA12 vs. HMA RCA21	2.45	1.92 × 10^−1^	No	6.61	6.20 × 10^−2^	No	50.37	2.08 × 10^−3^	Yes	4.77	9.43 × 10^−2^	No

**Table 7:** Results of the OAC for HMA.

Parameter	HMA Control	HMA RCA12	HMA RCA21	Requirement
OAC (%)	5.5	6.0	6.5	-
S (kN)	16.71	17.87	18.86	Minimum 9 kN
F (mm)	3.51	3.60	3.81	Between 2.0 and 3.5
S/F (kN/mm)	4.76	4.96	4.95	Between 3.0 and 6.0
Av (%)	4.21	5.40	5.70	Between 4.0 and 6.0
VMA (%)	16.54	18.50	19.69	Minimum 15
VFA (%)	74.55	70.83	71.11	Between 65 and 75

**Table 8:** ANOVA for the significance of RM test for HMA.

Analysis	STOA								
	2.5 Hz			5.0 Hz			10 Hz		
	F_T_	*p*-Value	Sig	F_T_	*p*-Value	Sig	F_T_	*p*-Value	Sig
Temperature 10 °C									
HMA Control vs. HMA RCA12	1.2	3.43 × 10^−1^	No	2	2.30 × 10^−1^	No	3	1.58 × 10^−1^	No
HMA Control vs. HMA RCA21	0.6	4.65 × 10^−1^	No	1.1	3.46 × 10^−1^	No	1.6	2.75 × 10^−1^	No
HMA RCA12 vs. HMA RCA21	0.1	8.30 × 10^−1^	No	0.1	7.39 × 10^−1^	No	0.7	4.63 × 10^−1^	No
Temperature 20 °C									
HMA Control vs. HMA RCA12	0.03	8.68 × 10^−1^	No	0.1	0.77 × 10^−1^	No	0.27	6.29 × 10^−1^	No
HMA Control vs. HMA RCA21	0.74	4.39 × 10^−1^	No	0.93	0.39 × 10^−1^	No	1.27	3.23 × 10^−1^	No
HMA RCA12 vs. HMA RCA21	0.37	5.75 × 10^−1^	No	0.23	0.65 × 10^−1^	No	0.09	7.79 × 10^−1^	No
Temperature 30 °C									
HMA Control vs. HMA RCA12	3.41	1.38 × 10^−1^	No	2.97	0.16 × 10^−1^	No	2.25	2.08 × 10^−1^	No
HMA Control vs. HMA RCA21	1.78	2.53 × 10^−1^	No	1.57	0.28 × 10^−1^	No	1.92	2.38 × 10^−1^	No
HMA RCA12 vs. HMA RCA21	22	9.35 × 10^−3^	Yes	18.1	0.013 × 10^−1^	Yes	17.8	1.35 × 10^−2^	Yes
Temperature 40 °C									
HMA Control vs. HMA RCA12	0.95	3.85 × 10^−1^	No	0.67	0.46 × 10^−1^	No	0.37	5.76 × 10^−1^	No
HMA Control vs. HMA RCA21	0.04	8.57 × 10^−1^	No	0.02	0.89 × 10^−1^	No	0.01	9.24 × 10^−1^	No
HMA RCA12 vs. HMA RCA21	0.55	5.01 × 10^−1^	No	0.36	0.58 × 10^−1^	No	0.19	6.88 × 10^−1^	No
**LTOA**									
Temperature 10 °C									
HMA Control vs. HMA RCA12	85.5	7.60 × 10^−4^	Yes	117	4.17 × 10^−4^	Yes	210	1.32 × 10^−4^	Yes
HMA Control vs. HMA RCA21	38.4	3.45 × 10^−3^	Yes	53.6	1.85 × 10^−3^	Yes	130	3.36 × 10^−4^	Yes
HMA RCA12 vs. HMA RCA21	1.44	2.96 × 10^−1^	No	1.19	3.36 × 10^−1^	No	0.08	7.87 × 10^−1^	No
Temperature 20 °C									
HMA Control vs. HMA RCA12	10.9	2.98 × 10^−2^	Yes	11.3	2.83 × 10^−2^	Yes	16.3	1.56 × 10^−2^	Yes
HMA Control vs. HMA RCA21	6.36	6.52 × 10^−2^	No	5.97	7.09 × 10^−2^	No	7.95	4.78 × 10^−2^	Yes
HMA RCA12 vs. HMA RCA21	0.65	4.65 × 10^−1^	No	0.52	5.11 × 10^−1^	No	0.36	5.81 × 10^−1^	No
Temperature 30 °C									
HMA Control vs. HMA RCA12	123	3.79 × 10^−4^	Yes	952	6.57 × 10^−6^	Yes	61.2	1.44 × 10^−3^	Yes
HMA Control vs. HMA RCA21	577	1.78 × 10^−5^	Yes	1127	4.69 × 10^−6^	Yes	346	4.91 × 10^−5^	Yes
HMA RCA12 vs. HMA RCA21	186	1.68 × 10^−4^	Yes	317	5.84 × 10^−5^	Yes	106	5.03 × 10^−4^	Yes
Temperature 40 °C									
HMA Control vs. HMA RCA12	29.1	5.73 × 10^−3^	Yes	32.8	4.60 × 10^−3^	Yes	27.7	6.24 × 10^−3^	Yes
HMA Control vs. HMA RCA21	43.8	2.70 × 10^−3^	Yes	44.4	2.64 × 10^−3^	Yes	35.2	4.05 × 10^−3^	Yes
HMA RCA12 vs. HMA RCA21	0.03	8.74 × 10^−1^	No	0.08	7.91 × 10^−1^	No	0.37	5.75 × 10^−1^	No

**Table 9:** dv (μm/min) and dv aging ratio for HMA.

Mixture	STOA	LTOA	Aging Ratio
HMA Control	4.47	2.26	0.51
HMA RCA12	2.39	3.44	1.44
HMA RCA21	4.05	3.81	0.94

**Table 10:** ANOVA for the significance of final displacement test for HMA.

Analysis	STOA			LTOA		
	FT	*p*-Value	Sig	FT	*p*-Value	Sig
HMA control vs. HMA RCA12	11.1	2.90 × 10^−2^	Yes	19.6	1.14 × 10^−2^	Yes
HMA control vs. HMA RCA21	23.4	8.44 × 10^−3^	Yes	64.2	1.31 × 10^−3^	Yes
HMA RCA12 vs. HMA RCA21	56.2	1.69 × 10^−3^	Yes	200.8	1.44 × 10^−4^	Yes

**Table 11:** ANOVA for the significance of ITSdry and ITSwet for HMA.

Analysis	STOA			LTOA		
	F_T_	*p*-Value	Sig	F_T_	*p*-Value	Sig
**Dry**						
HMA Control vs. HMA RCA12	5.7	7.60 × 10^−2^	No	7.8	4.93 × 10^−2^	Yes
HMA Control vs. HMA RCA21	2.5	1.89 × 10^−1^	No	0.0004	9.84 × 10^−1^	No
HMA RCA12 vs. HMA RCA21	0.1	7.49 × 10^−1^	No	23.7	8.24 × 10^−3^	Yes
**Wet**						
HMA Control vs. HMA RCA12	1.5	2.87 × 10^−1^	No	27.1	6.48 × 10^−3^	Yes
HMA Control vs. HMA RCA21	3.6	1.30 × 10^−1^	No	2.7	1.73 × 10^−1^	No
HMA RCA12 vs. HMA RCA21	0.02	8.95 × 10^−1^	No	1.9	2.38 × 10^−1^	No

**Table 12:** Classification of HMA for Marshall’s test, RM, and rutting resistance.

Type Mixture	Marshall’s Test						RM												Maximum Displacement			dv		
	S			S/F			10 °C			20 °C			30 °C			40 °C								
	STOA	LTOA	Aging Ratio	STOA	LTOA	Aging Ratio	STOA	LTOA	Aging Ratio	STOA	LTOA	Aging Ratio	STOA	LTOA	Aging Ratio	STOA	LTOA	Aging Ratio	STOA	LTOA	Aging Ratio	STOA	LTOA	Aging Ratio
HMA Control	3	3	1	3	1	1	1	1	1	1	1	1	2	1	1	2	1	1	2	1	1	3	1	1
HMA RCA12	2	2	2	1	3	3	2	3	3	2	3	3	1	3	3	1	2	3	1	2	3	1	2	3
HMA RCA21	1	1	3	2	2	2	3	2	2	3	2	2	3	2	2	3	3	2	3	3	2	2	3	2

**Table 13:** Classification of HMA for IDT and TSR.

Type Mixture	IDT						TSR		
	ITS Dry			ITS Wet					
	STOA	LTOA	Aging Ratio	STOA	LTOA	Aging Ratio	STOA	LTOA	Aging Ratio
HMA Control	1	1	2	1	1	1	3	1	1
HMA RCA12	3	3	3	2	3	3	1	2	3
HMA RCA21	2	2	1	3	2	2	2	3	2

**Table 14:** COVs for mixtures results.

Type Mixture	Marshall Test		RM												Maximum Displacement	IDT	
			10 °C			20 °C			30 °C			40 °C				ITS Dry	ITS Wet
	S	S/F	2.5 Hz	5 Hz	10 Hz	2.5 Hz	5 Hz	10 Hz	2.5 Hz	5 Hz	10 Hz	2.5 Hz	5 Hz	10 Hz			
**STOA**																	
HMA Control	3.2	4.6	9.5	8.6	9.6	8.5	7.8	7.0	9.9	9.8	9.3	8.0	8.4	8.4	4.2	6.6	4.4
HMA RCA12	5.5	6.5	2.7	3.4	3.9	8.6	8.5	8.2	5.7	5.8	5.9	15.5	14.4	12.7	6.8	4.1	13.1
HMA RCA21	2.7	2.7	6.4	5.6	4.6	1.6	0.5	0.3	6.9	7.1	6.3	28.4	26.6	25.5	1.7	7.8	6.2
**LTOA**																	
HMA Control	4.3	5.8	2.6	2.1	1.5	4.2	4.3	3.5	2.5	2.0	1.8	7.4	7.4	8.3	18.2	5.8	2.7
HMA RCA12	4.6	7.5	1.9	1.6	1.1	6.3	5.8	4.9	0.8	0.9	1.7	7.1	5.7	4.8	0.7	3.1	5.7
HMA RCA21	3.6	2.1	3.4	2.9	1.9	5.7	6.0	6.0	3.7	2.9	2.0	1.5	1.5	1.4	2.6	2.6	9.4

## Data Availability

The original contributions presented in this study are included in the article. Further inquiries can be directed to the corresponding author.
